# Supplementary figures and images for: Luminal and Mucosal Microbiota of the Cecum and Large Colon of Healthy and Diarrheic Horses
Source: Animals (Basel). 2020 Aug 12;10(8):1403. doi: 10.3390/ani10081403 (PMC7460328; doi:10.3390/ani10081403)

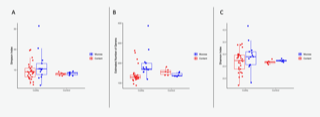

Supplement: Supplementary file 1 [file animals-10-01403-s001.zip › animals-850072-Figure S1-s3/Supplementar Figure 1 Alpha diversity.tiff]

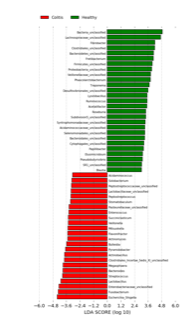

Supplement: Supplementary file 1 [file animals-10-01403-s001.zip › animals-850072-Figure S1-s3/Supplementary figure 2 LEfSe LDA 2.tiff]

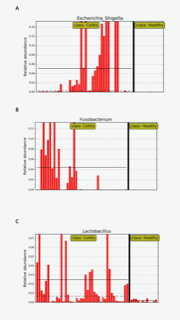

Supplement: Supplementary file 1 [file animals-10-01403-s001.zip › animals-850072-Figure S1-s3/Supplementary figure 3.tiff]
